# Flexible protein database based on amino acid k-mers

**DOI:** 10.1038/s41598-022-12843-9

**Published:** 2022-06-01

**Authors:** Maxime Déraspe, Sébastien Boisvert, François Laviolette, Paul H Roy, Jacques Corbeil

**Affiliations:** 1 Department of Molecular Medicine, Université Laval, Quebec, Canada; 2 Big Data Research Center, Université Laval, Quebec, Canada; 3 Bodycad, 2035 rue du Haut-Bord, Quebec, Canada; 4 Infectious Disease Research Centre, Université Laval, Quebec, Canada; 5 Department of Computer Science, Université Laval, Quebec, Canada; 6 Centre NUTRISS, Université Laval, Quebec, Canada

**Keywords:** Antimicrobials, Bacteria, Scientific data, Genomic analysis, Software, Sequence annotation

## Abstract

Identification of proteins is one of the most computationally intensive steps in genomics studies. It usually relies on aligners that do not accommodate rich information on proteins and require additional pipelining steps for protein identification. We introduce kAAmer, a protein database engine based on amino-acid k-mers that provides efficient identification of proteins while supporting the incorporation of flexible annotations on these proteins. Moreover, the database is built to be used as a microservice, to be hosted and queried remotely.

## Introduction

One fundamental task in genomics is the identification and annotation of DNA coding regions that translate into proteins via a genetic code. Protein databases increase in size as new variants, orthologous and novel genes, often found in metagenomics studies, are being sequenced. This is particularly true within the microbial world where bacterial proteomes’ diversity follows their rapid evolution. For instance, UniProtKB (Swiss-Prot/TrEMBL)^1^ and NCBI RefSeq^2^ contain over 100 million bacterial proteins and that number is increasing rapidly.

Identification of proteins often relies on accurate, but slow, alignment software such as BLAST or hidden Markov model (HMM) profile-based software^3,4^. Although other approaches (such as DIAMOND^5^) have considerably improved the speed of searching proteins in large datasets, from a database standpoint much can be done to offer a more versatile experience. One such approach would be to expose the database as a permanent service, which can make use of computational resources for increased performance (e.g. memory mapping) and leveraging the cloud for remote analyses via a HTTP API. Another approach would be to extend the result set with comprehensive information on protein targets to facilitate subsequent genomics and metagenomics analysis pipelines.

Alignment software typically relies on a seed-and-extend pattern using an index (two-way indexing in DIAMOND) to make local alignments between query and target sequences. However, there is a plethora of research techniques to bypass the computational cost of alignment. Alignment-free sequence analyses usually adopt k-mers (overlapping subsequences of length k) as the main element of quantification. They are extensively used in DNA sequence analyses ranging from genome assemblies^6^ to genotyping variants^7^, as well as genomics and metagenomics classification^8–10^. In the present study, we introduce kAAmer, a fast and comprehensive protein database engine that was named after the usage of amino acid k-mers (kAAmer: *k-amino-acid-mer*) which differs from the usual nucleic acid k-mers. We demonstrate the usefulness and efficiency of our approach in protein identification with a protein domain database and antibiotic resistance gene identification from a pan-resistant bacterial genome.

## Results and discussion

**Figure 1:**
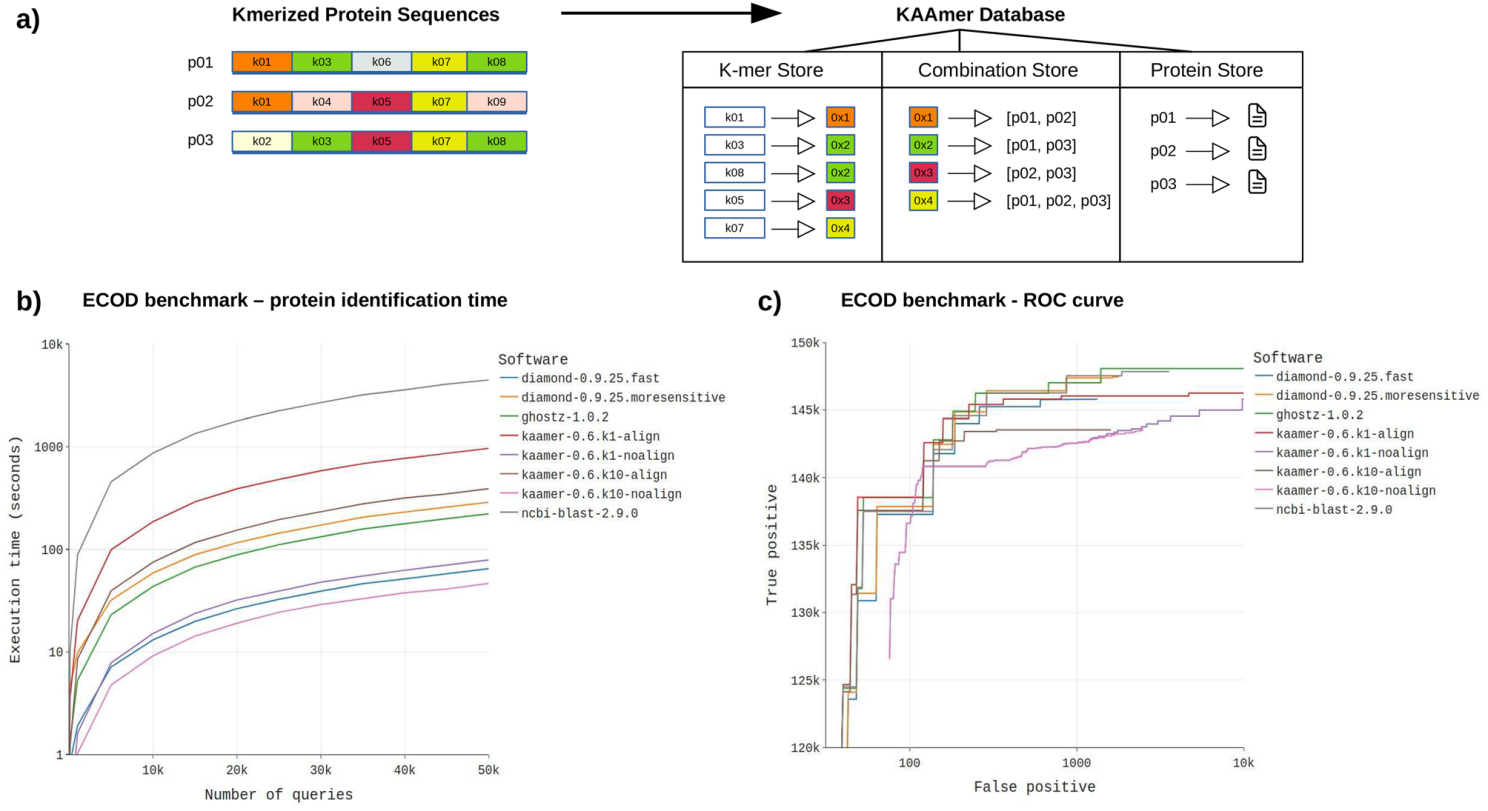
(**a**) Design of a kAAmer database. Three key-value stores are created within a database (K-mer Store, Combination Store, Protein Store). Colours indicate the combination (hash) values that are reused in the combination store. Proteins are numbered (p01, p02, p03) and k-mers are numbered (k01, k02, ..., k08). (**b**) Protein search speed benchmark. Software include Blastp (v2.9.0+), Ghostz (v1.0.2), Diamond (v0.9.25) and kAAmer (v0.6) with (-aln) and without (-kmatch) alignment. (**c**) Protein search precision and recall benchmark with the ECOD database. The blue bars indicate the precision results and the red bars indicate the recall results. Software include Blastp (v2.9.0+), Ghostz (v1.0.2), Diamond (v0.9.25) and kAAmer (v0.6) with alignment.

The database engine of kAAmer is based on log-structured merge-tree (LSM-tree) Key-Value (KV) stores^11^. LSM-trees are used in data-intensive operations such as web indexing^12,13^, social networking^14^ and online gaming^15,16^. KAAmer uses Badger^17^, an efficient implementation in Golang (https://golang.org/) of a WiscKey KV (key-value) store^16^. WiscKey’s LSM-tree design is optimized for solid state drives (SSD) and separates keys from values to minimize data movement during the creation of the key-value store. KAAmer will obtain peak performance with modern hardware such as solid-state drives that offer good throughput in input/output (I/O) operations per second (IOPS) and will effectively accommodate use cases where many queries are sent simultaneously. A kAAmer database includes three KV stores (see Fig. 1a): one to provide the information on proteins (protein store) and two to enable the search functionalities (k-mer store and combination store). The k-mer store contains all the 7-mers found in the sequence dataset and the keys to the combination store, which uniquely serves the combination of proteins held by k-mers. The fixed k-mer size of 7 was chosen to fit on 4 bytes and keep a manageable database size while offering good specificity over protein targets. The k-merized design of a kAAmer database provides an interesting simplicity for the search tasks which will give an exact match count of all 7-mers between a protein query and all targets from a protein database. This strategy is not guaranteed to return the same homologous targets that would be obtained with alignment or HMM search and is therefore less suitable for distant homology retrieval.

In order to evaluate the performance and precision of kAAmer, we built a speed and a sensitivity benchmark against protein families of the ECOD database (homology groups)^18^. We evaluated four software: Blastp (v2.9.0+)^3^, Ghostz (v1.0.2)^19^, Diamond (v0.9.25)^5^ and kAAmer (v0.6). Other interesting software that make use of web servers for remote analyses, such as Sequenceserver^20^ and MMseqs2^21^, are worth mentioning for the functionality that they offer. However, we limited our benchmark to the previously mentioned software for their alignment efficiency and their computational resource requirements. Note that we have tested two modes for Diamond, the more-sensitive and fast ones. Similarly, we tested two sensitivity modes with kAAmer, based on the minimal number of shared k-mers, which are k10 (at least 10 shared k-mers between protein query and target) and k1 (at least 1 shared k-mer). For kAAmer, each sensitivity mode was tested without alignment—the ratio of shared k-mers serving as a scoring function and also with a subsequent alignment form. The alignment purpose in kAAmer is to improve the scoring metrics while using the same result set as the raw alignment-free method.

Figure 1c illustrates the ROC curve results of the sensitivity benchmark. We observed that Ghostz, Blastp and Diamond-sensitive reported respectively the highest number of true positives regardless of false negatives. Then follows, kAAmer with the minimal number of shared k-mers, Diamond-fast and kAAmer with at least 10 shared k-mers. The ROC curve also shows the difference in precision of the alignment-free mode of kAAmer compared to the alignment modes. One of the main reasons would be the scoring scheme that uses the percentage of shared k-mers in contrast to bit score with the alignment results. Note that the minimal k-mer matches is an option provided by the user to tune the sensitivity of the protein search. We also compared our database engine with the aforementioned software for their execution time with different query dataset size. Thirteen different protein query datasets were randomly and uniquely chosen from the original ECOD database, with size ranging from 1 protein to 50,000 proteins. Figure 1b illustrates the wallclock times of the alignment software in comparison with kAAmer for protein homology searches. See the Methods section for the hardware used in the benchmarks. We observe with the larger query datasets (50,000 proteins) that kAAmer k10 in alignment-free mode completed the search in 46.5 s, while the alignment mode for kAAmer-k10 did it in 390.7 s. When using only one shared k-mers (kAAmer k1), the most sensitive mode in kAAmer, the execution times were 78.8 s without alignment and 966.6 s with alignment. The fast mode of Diamond completed the same task in 64.7 s, while it took 287.7 s with the sensitive mode. Ghost yielded results similar to the Diamond sensitive mode while Blastp reported results that were significantly slower than the other tested software. When comparing the speed results with the maximum number of queries (50,000 proteins), kAAmer in its alignment-free mode achieves performance comparable to the fast mode of Diamond, although the results will vary with the parameter of the minimal number of k-mer matches used. The alignment mode of kAAmer obviously adds an overhead that will impact the running time results. Yet in combination with the minimal k-mer match of 1, it will offer better sensitivity at the detriment of speed.

In order to accomodate real-use cases, we built relevant kAAmer databases and investigated their usage in typical bacterial genomics analyses. It should be noted that annotation of genomes and gene identification rely heavily on the quality of the underlying database. What kAAmer has to offer is the inclusion of the protein information within the database combined with an efficient search functionality to facilitate downstream analyses. Therefore, we also provide utility scripts to demonstrate these use cases. The first use case was to identify antibiotic resistance genes (ARGs) in a bacterial genome and test its accuracy related to other ARG finder software. For ARG identification we used the NCBI Bacterial Antimicrobial Resistance Reference Gene Database (v2020-01-06.1)^22^ and compared the kAAmer results with the ResFinder (v3.2 and database 2019-10-01)^23^ and CARD (v5.1.0)^24^ software and database. The query genome is a pan-resistant *Pseudomonas aeruginosa* strain E6130952^25^. Table 1 shows the results of the ARG identification within the query genome by the three software / databases tested. For the majority of antibiotic classes, the results are in agreement between the three databases. Interestingly, three aminoglycoside genes (*aac(6’)-Il*, *ant(2”)-Ia* and *aacA8*) were only found with kAAmer (NCBI-ARG) and ResFinder. On the other hand, several more antibiotic efflux systems are annotated in CARD and the number of identified efflux proteins in E6130952 goes up to 36 while only 3 were reported by kAAmer (NCBI-ARG) and none by ResFinder. Also 2 genes associated with resistance to peptide antibiotics (arnA, basS) and 2 other (soxR, carA) associated with multiple antibiotic classes were only reported by CARD. Other tested use cases include genome annotation and metagenome profiling as shown in the Methods section.

**Table 1:** Report of the antibiotic resistance gene identification in the pan-resistant Pseudomonas aeruginosa E6130952 strain from kAAmer+NCBI-arg, ResFinder and CARD databases.

Resistance gene	Antibiotic class	kAAmer+NCBI-ARG	ResFinder	CARD
*aac(6’)-Il*	Amikacin/kanamycin/tobramycin	3	3	0
*ant(2”)-Ia*	Gentamicin/kanamycin/tobramycin	1	1	0
*aacA8*	Aminoglycoside	1	1 (*aac(6’)-31*)	0
*aph(3’)-IIb*	Kanamycin	1	1	1
*aadA6*	Streptomycin	2	2	2
$\textit{bla}_{\mathrm{OXA}\text {-}2}$, $\textit{bla}_{\mathrm{OXA}\text {-}488}$	Beta-lactam	2	2	2
$\textit{bla}_{\mathrm{PDC}\text {-}35}$	Cephalosporin	1	1 ($\textit{bla}_{\mathrm{PAO}}$)	1 ($\textit{bla}_{\mathrm{PDC}\text {-}2}$)
*fosA*	Fosfomycin	1	1	1
*catB7*	Chloramphenicol	1	1	1
*sul1*	Sulfonamide	3	3	3
*mexA*, *mexE*, *mexX*	Efflux	3	0	2 (no *mexX*)
Other efflux system	Efflux	0	0	34
*arnA*, *basS*	Peptide antibiotic	0	0	2
*soxR*, *carA*	Multiple antibiotic class	0	0	2
Total	13	19	16	51

In summary, kAAmer introduces a fast and flexible protein database engine to accommodate different genomics analyses use cases. It can be hosted on-premise or in the cloud and be queried remotely while offering a flexible protein annotation scheme. Although it can be adapted to find more distant homology, it is best suited to quickly find close sequence homology with its k-mer matching functionality, while providing rich annotations on the identified protein targets.

## Methods

### Design of kAAmer

KAAmer design was influenced by our requirements that protein databases would be permanently hosted (on premise or in the cloud), queried remotely and would have room to scale as sequence databases grow in size. It also needed to be multithreaded for protein searches and would support alignment for more accurate remote homology findings. We opted for a Key-Value store engine that would reside on disk and be optimized for SSDs. We used the Go programming language for its versatility and efficiency. The Key-Value stores use the Badger^17^ engine and protein annotations are encoded using Protocol Buffers^26^.

### Database building

KAAmer is first used to build a database in which all amino acid k-mers are associated with proteins in which they are found. It consists of three KV stores to hold the database information (k-mer store, combination store and protein store). The first KV store (k-mer store) keeps the association of every k-mer (key) with a hash value (key length: 8 bytes) that is the entry to the combination store. The k-mer size is fixed at 7 amino acids to fit k-mer keys onto 32 bits (4 bytes) and thus maintain a manageable final database size while keeping a k-mer size long enough for specificity. The second KV store (combination store) is used to hold all the unique sets of protein identifiers. The method used to build this store can relate to the flyweight design pattern or the hash consign technique. Indeed, hash values are reused to access identical objects and therefore minimize memory usage. The set of protein identifiers are the keys to the third store (protein store) which contains the protein information found in the raw annotation file. The raw input file can be either in the EMBL format, GenBank format, TSV format or in FASTA format.

### Querying a database

Once we have a database, we expose it with the kAAmer server that listens over HTTP for incoming requests. The benefits of using such a service are two fold. First, the database is opened once and is memory mapped to increase the performance of protein searches. Second, the kAAmer server can be hosted virtually anywhere, in the cloud for instance, and be queried remotely by the kAAmer client. Note that it is preferable that the latency (time required for a message to be transported over HTTP) between the server and client be as low as possible. KAAmer supports protein query and translated DNA query from FASTA input as well as short reads sequences (like Illumina) in FASTQ format. The default mode in kAAmer finds and reports k-mer exact matches (“kmatch”) with target proteins from the database. However, kAAmer also provides an alignment mode (“kaamer-aln”) that produces protein sequence alignments on the k-mer matches’ result set with the Smith-Waterman algorithm, as implemented in the biogo package^27^. Options for the alignment mode include the substitution matrix and the gap open and gap extend penalty values. The user can also control the minimal number of k-mer matches to report a hit as well as the minimal ratio of k-mer matches over the protein queries.

### ECOD database benchmarks

The ECOD database contains 149,091 protein sequences classified into 88 homology groups. The sensitivity benchmark is based on those homology groups. For each homology group, we identify all the homology pairs where the query is effectively part of the analyzed group. A true positive occurs when the subject of the detected homology belongs to the same group of the query and a false positive when it belongs to a different group. Each protein can only be identified once from the query set and it must not be the same protein that reports the homology, therefore the maximum number of true positives is equal to the size of the database. The ROC curve is built by changing the score threshold (bit score or number of shared k-mers) and by counting the number of true and false positives respecting it^28^.

For the speed benchmark on the ECOD proteins database, we randomly and uniquely extracted multiple sets of sequences, with the number of sequences ranging from 1 to 50,000. Each set of sequences was in its own FASTA file to be queried against the whole database with the different alignment software included in the benchmark. The benchmark for all four software (Blastp (v2.9.0+), Ghostz (v1.0.2), Diamond (v0.9.25) and kAAmer (v0.6)) was run on nodes geared with 32 cores (2nd generation AMD EPYC Processor), 64 GB of RAM and with a NVMe connected SSD (Amazon EC2 c5ad.8xlarge instance). Software were run with default parameters, except for the number of threads set to 32 and the maximum number of results equal to the total number of proteins in the database. We should note that Diamond yields faster results with large query sets, however, comparison purposes of speed and sensitivity both benchmarks used the same ECOD dataset. More details on the benchmark is provided at https://github.com/zorino/kaamer-benchmark.

### Other kAAmer use cases

Apart from the antibiotic resistance gene (ARG) identification use case, we also provide two demonstrations of kAAmer usage in bacterial genome annotation and metagenome profiling. The use cases are documented at https://github.com/zorino/kaamer_analyses and a Python script is provided for each one of the analyses. For the genome annotation, we used the chromosomal sequence of the same *Pseudomonas aeruginosa* strain (E6130952) as in the antibiotic resistance genes identification. The kAAmer database that was used for the homology detection is a subset of RefSeq from the *Pseudomonadaceae* family which is available from the kAAmer repository (see Data availability) along with other bacterial family databases. Essentially the genome annotation script parses the kAAmer results and produces a GFF (General Feature Format) annotation file giving some threshold on the protein homology. The other use case is the profiling of a metagenome based on the MGnify database of the human gut^29^. MGnify includes protein annotations from gene ontology, enzyme commission and kegg pathways, among others. The metagenome profiling script will parse the results and produce a summary file by annotation that counts the presence and abundance of each feature.

## Data Availability

The kAAmer software is available under the Apache Version 2.0 license at https://github.com/zorino/kaamer. The documentation is available at https://zorino.github.io/kaamer/. Several pre-built kAAmer databases are available at https://kaamer.genome.ulaval.ca/kaamer-repo/.
